# 1353. Incidence of Lyme Borreliosis in Germany: Exploring Observed Trends Over Time Using Public Surveillance Data, 2016–2020.

**DOI:** 10.1093/ofid/ofac492.1182

**Published:** 2022-12-15

**Authors:** Jozica Skufca, Thao Mai Phuong Tran, Gordon Brestrich, Andreas Pilz, Andrew Vyse, Claudius Malerczyk, Mendwas Dzingina, Elizabeth Begier, Maxim Blum, Margarita Riera, Bradford Gessner, James Stark

**Affiliations:** P95, Leuven, Vlaams-Brabant, Belgium; P95, Leuven, Vlaams-Brabant, Belgium; Pfizer Vaccines, Berlin, Berlin, Germany; Pfizer Corporation Austria, Vienna, Wien, Austria; Pfizer UK, Tadworth, England, United Kingdom; Pfizer Vaccines, Berlin, Berlin, Germany; Pfizer UK, Tadworth, England, United Kingdom; Pfizer Vaccines, Berlin, Berlin, Germany; P95, Leuven, Vlaams-Brabant, Belgium; P95, Leuven, Vlaams-Brabant, Belgium; Pfizer US, New York, New York; Pfizer US, New York, New York

## Abstract

**Background:**

Lyme borreliosis (LB) is an infectious, vector-borne disease caused by the spirochete *Borrelia burgdorferi* sensu lato and mainly transmitted by the *Ixodes* species ticks. Public surveillance of LB occurs in 9 of 16 federal states of Germany and remains a critical facet of disease epidemiology and trends. We describe the incidence, time trends, seasonality, and geographic distribution of LB in Germany using publicly reported surveillance data.

**Methods:**

We obtained LB cases and incidence (2016-2020) from the online platform SurvStat@RKI 2.0, maintained by the Robert Koch Institute (RKI). Cases and incidence of LB were extracted by year, geographic area, week of notification, sex, age and by 2 case definition categories (clinically diagnosed LB, clinically diagnosed and laboratory-confirmed LB) reported by 9 states where LB notification is mandatory. The nine states include Bavaria, Berlin, Brandenburg, Mecklenburg-Vorpommern, Saxony, Saxony-Anhalt, Thuringia, Rhineland-Palatinate, and Saarland.

**Results:**

During 2016-2020, the 9 federal states reported 63,940 LB cases, of which 60,570 (94.7%) were clinically diagnosed, and 3,370 (5.3%) also had laboratory confirmation, with an average of 12,789 cases annually. LB incidence rates were mostly stable over time. The average annual LB incidence was 37.2/100,000 person-years, ranging from 22.9 to 64.6/100,000 person-years among 9 states; from 16.8 to 85.6/100,000 person-years among 19 regions; and from 2.9 to 172.8/100,000 person-years among 158 counties. Incidence was lowest among persons 20‒24 years old (16.1/100,000 person-years) and highest among those aged 65‒69 years old (60.9/100,000 person-years). Most cases were reported between June and September, with a peak in July of every year.

Annual incidence (per 100,000 person-years) of overall Lyme borreliosis notified in 158 counties (NUTS3), 2016-2020

**Figure:**
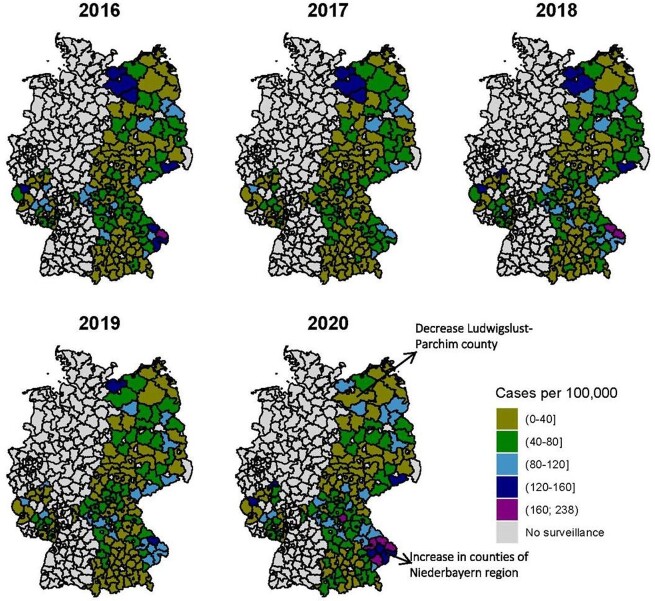

**Conclusion:**

The risk of LB varied substantially at the smallest geographic unit and to a lesser degree by age group. Our results underscore the importance of presenting LB data at the most spatially granular unit and by age to allow implementation of efficient preventive interventions and reduction strategies.

**Disclosures:**

**Jozica Skufca, Epidemiologist**, p95: Paid by Pfizer to perform the study **Thao Mai Phuong Tran, Ph.D.**, P95: P95 was paid by Pfizer to perform the study **Gordon Brestrich, PhD**, Pfizer: Employee|Pfizer: Stocks/Bonds **Andreas Pilz, PhD**, Pfizer: Employee|Pfizer: Stocks/Bonds|Pfizer: Stocks/Bonds **Andrew Vyse, Ph.D.**, Pfizer: Stocks/Bonds **Claudius Malerczyk, PhD**, Pfizer: Employee|Pfizer: Stocks/Bonds **Mendwas Dzingina, Ph.D.**, Pfizer: Stocks/Bonds **Elizabeth Begier, M.D., M.P.H.**, Pfizer: Employee|Pfizer: Stocks/Bonds **Maxim Blum, Ph.D.**, P95: Paid by Pfizer to perform the study **Margarita Riera, MD, MPH**, P95: Paid by Pfizer to perform the study **Bradford Gessner, MD, MPH**, Pfizer: Stocks/Bonds **James Stark, Ph.D.**, Pfizer: Stocks/Bonds.

